# Progress in Surgery

**Published:** 1899-09-02

**Authors:** 


### Progress in Surgery.

### APPENDICITIS.

Edebohls has recently published his experiences of the association and co-existence of appendicitis and floating kidney. One would imagine that he imbued his patients with disease, for one patient was exceedingly complex, requiring the fixation of both kidneys, the removal of the appendix, curettage of the uterus, and excision of hæmorrhoids. He says that after he had learned to diagnosticate chronic appendicitis the frequent association of that condition with symptom-producing right movable kidney, the apparent dependence of the former upon the latter, and the important part played by the appendix in gynæcological matters arrested his attention. He finally found himself in a position fully and permanently to cure by surgical measures almost every patient who came to him as a proper subject for gynæcological treatment. In his experience chronic appendicitis is present in 90 per cent. of cases of movable right kidney, and 20 per cent. of all women have movable kidneys. Chronic appendicitis, he thinks, may be the only symptom of movable right kidney, and they are directly connected as cause and effect. In some cases right nephropexy will cure the appendicitis, but the majority require appendicectomy at the same time. The multiplicity of operations requires two sittings.

Edebohls thinks that no examination of the pelvic viscera should be regarded as complete that does not note the appendix. Similarly in all cases of cœliotomy for pelvic disease the appendix should be investigated, removed if diseased, and inverted if normal! Although few will follow the author in his extent of operative treatment, many recognise the marked relationships between renal and pelvic morbid states.

Our Parisian neighbours have been again much concerned with the question as to whether appendicitis is medical or surgical. The Society of Surgery seems to have arrived at the opinion that there is no medical treatment for appendicitis.[2] The affection is usually due to infection by the bacillus coli or by streptococci. The coats of the appendix become invaded in order, till finally the peritoneum is reached and a plastic or suppurative peritonitis results. It appears that the blood vessels of the appendix possess few anastomoses in their terminal ramifications, so that anæmic infarcts and necrotic areas are very prone to result. The scantiest blood supply is towards the proximal end, and it is here that the perforation is more prone to occur. If a spontaneously favourable termination occurs the abscess probably perforates into the intestine.[3]

The question when to operate for appendicitis is differently answered by different surgeons. However, much depends upon the definition of the word. Some exclude a catarrhal inflammation of the mucosa, and only include infective processes of the walls of the appendix. Dr. Robert T. Morris has studied the question, and, on the whole, urges early operation. He says, "the reason why we are to operate as soon as the diagnosis of appendicitis has been made is that the surgical death-rate is lower than the medical death-rate, if the surgical work is properly done." He adds also the diminished "suffering-rate" and the lessened "time-rate." When the inflammation is limited to the walls of the appendix the death-rate should not be higher than 1 per cent. In the virulent septic cases Morris holds that several methods of procedure have a distinct death-rate of their own, and that by excluding them he has been able to reduce his mortality. As these are personal experiences of some value, it may be well to consider them seriatim.

Iodoform gauze packing he holds to be injurious, for two reasons. One is the great rapidity with which iodoform poisoning may occur from gauze placed in the peritoneal cavity, although the wound may look perfectly healthy. Moreover, mere gauze packing is injurious. It causes shock, it causes excessive exudation of putrescible lymph, and ultimately troublesome adhesions. It is painful to remove, and may cause ileus by its mechanical effect. Then, again, the risk of post-operative ventral hernia is greatly increased by gauze packing. In place of this Morris recommends a small wick covered with gutta-percha tissue to avoid formation of adhesions.

The risk of leaving an infected appendix among adhesions is great, and should not be necessary if surgeons are accustomed to work among adhesions.

He characterises as a "dreadful method" the plan of making long incisions and of partial evisceration of the patient, under the imagined necessity of cleansing pus and lymph from loops of bowel. In advanced septic infection of the peritoneum the duty of the surgeon is to get in quickly, to get out quickly, and to be gentle.[4] Dr. A. B. Johnson, however, thought long incisions were advantageous, affording facilities for detecting small abscesses; moreover, the mechanical effect of iodoform gauze was much more serious than the risks of iodoform poisoning. He records an experiment where gauze had been packed amongst the coils of intestines as a "precautionary" measure. Forty-eight hours afterwards the wound had to be opened for intestinal obstruction, when the intestine close to the strings of gauze had become so dark in colour, and so evidently paralysed, that the intestine was opened and an artificial anus established, thus saving the patient's life. Dr. Parker Syms agreed with Dr. Morris in urging operation as soon as a diagnosis of appendicitis was made.

The question of the time to operate has also been discussed in France.[5] Many surgeons adopted the American view, that it is best to operate at once on all cases of appendicitis from ignorance of the possible fatal complications. Schwartz, the reader of the paper, advocated expectant treatment in acute attacks, deferring operation unless there were signs of commencing peritonitis. Quenu and Tuffier were in favour of immediate operation; others in favour of waiting until the area was walled off by peritonitic adhesions.

Richardson and Brewster have given the results of personal observations and treatment of 750 cases of appendicitis.[6] Of 464 acute cases, 284 were operated on with a mortality of 21 per cent., and 149 cases recovered without operation. All the cases operated on in the interval recovered, and these amounted to no less than 151. General infection is commonest on the third or fourth day, and it is then that the question of operation should be very carefully considered. Cultures of the surrounding fluids will show whether they are infected or not. Operative success usually depended on the localising of the abscess by adhesions. Local abscesses of a week or more duration all recovered. Of 180 cases treated medically only 17 per cent. died, a lower mortality than after operation, but some of the fatal cases might have been saved by operation. To summarise, note the safety of interval operations, and the necessity for consulting the surgeon at an early stage.

In connection with appendicitis the two cases recently recorded by Mr. Mansell Moullin and his colleagues are interesting. Reference may be made to the figure illustrating the rare condition of hernia iliaco-subfascialis, *i.e.*, hernia into a peritoneal pouch upon the iliacus muscle, external to the iliac artery and internal to the cæcum.[7]

Kammerer, Jalaguier, Lennander, Battle, and others have advocated operation upon the appendix by valvular incision, deflecting the right rectus muscle to the middle line.[8] The chief disadvantage is, perhaps, the possible division of one or more of the fibres to the rectus muscle with consequent atrophy. Kammerer records and details 18 cases treated by this method.

Anomalous conditions associated with the appendix are always appearing. Mr. George Heaton records three such cases; one with an acute strangulation of the tip of an appendix in a retroperitoneal pouch; a second with the appendix acutely kinked and the terminal end dilated and bulbous; and a third with a chronically inflamed appendix adherent to the iliac vessels and fixed in the true pelvis.[9]

Dr. G. E. Brewer gives four atypical cases, one with an inflamed appendix concealed in a subcæcal fossa, another with an extra-peritoneal retrocæcal appendix, another succeeded by fatal intestinal obstruction, and a fourth by peritoneal abscess.

[1] New York Med. Rec., Mar. 11, 1899. [2] Lancet, May 13, 1899. [3] New York Med. Jour., Nov. 12, 1898. [4] New York Med. Jour., April 29, 1899. [5] Med. News, Mar. 11, 1899. [6] Epit. Brit. Med. Jour., Dec. 17, 1898. [9] Lancet, May 13, 1899. [8] Med. Rec., June 10, 1899. [7] Birmingham Med. Rev., June, 1899.

### ORTHOPÆDIC SURGERY.

**Forcible Rectification of Spine.**—Lange,[1] of Munich, considers that Calot's treatment of spinal caries by forcible straightening is open to serious objection in most cases. He states that early results in the majority of cases are promising, but the improvement supposed to be gained does not last. The large gap formed by separating the remains of the diseased vertebræ is occupied at first by blood or pus, and, subsequently, either by tuberculous granulations in weakly subjects, or, in the most favourable conditions, by fibrous tissue. The author thinks that it has still to be proved that such gap is ever occupied by new bone. The straightened spine may acquire a certain degree of stability in consequence of osseous fusion of the arches and processes of the diseased vertebræ, but the back will always remain very weak at the seat of the old disease and be subject to fracture from slight injury, and to the risk in such fracture of serious and perhaps fatal compression of the cord. The condition of a patient whose spine has been straightened with apparently good results may be compared to that of one who has lost a part of the bone of the skull. The only class of cases that Lange considers call for forcible straightening are those where angular curvature is associated with persistent paralysis of the lower limbs, and in these cases it is worth while to run the risks in order to relieve the patient of a very distressing condition.

Dr. L. Wullstein,[2] of Halle, gives three cases of Ménard's of operation on the diseased cadaver. There was a gap created of from three to six to eight centimetres between the upper and lower segments of the spine; in one case an abscess was ruptured, and made to communicate with the mediastinum; in no case was there any injury to the spinal cord or its membranes. Von Brun caused a gap in a spine of fully eight centimetres. Vulpins found a gap of several centimetres in the line of the vertebral bodies where there was most destruction, viz., the tenth and eleventh dorsal, in a child who died forty-eight hours after forcible rectification. The author operated in two cases. A male, aged 23, in whom disease began at four years of age, had temporary paralysis of the lower extremities, bladder, and rectum before his sixth year, and kyphosis of more than half the dorsal and lumbar vertebræ. Under uniform and gradually increasing traction most of the deformity disappeared with a crackling sound. When the deformity had been completely corrected under much direct pressure the diastasis was fully nine centimetres wide. The patient died from pyelonephritis. In the region of the diastasis the vertebral bodies had almost disappeared; everywhere the dura was rigid, thickened, and partly calcified: The spine was fractured between the ninth and tenth spinous processes, and it contained old and new foci of tuberculous disease from the first dorsal to the fourth lumbar vertebræ. Above the diastasis there was a large abscess and another below it, but neither of these abscesses were ruptured during the operation. The second case was a female, aged six years, in whom there was temporary paralysis of the lower extremities. Death was caused by pulmonary tuberculosis. Correction was easy, and examination showed two diastases, the upper being four centimetres and the lower three centimetres. In the upper the dura was exposed and rendered exceedingly tense, but was uninjured. About five centimetres to the left of the upper diastasis was an unruptured abscess, and another was found in the right psoas muscle. In this diastasis there were the remains of an abscess capsule, which must have been divided during the operation. The fifth and sixth dorsal vertebræ were totally, the fourth and seventh partially, destroyed. In the lower diastasis the third and fourth lumbar vertebræ were partly destroyed, and the left half of the fifth entirely. The posterior half of the spine was completely fractured between the spinous processes of the fifth and sixth dorsal vertebræ. Between the sixth and seventh vertebral arches the softened spinal cord was so torn and stretched that it was with difficulty removed without being completely torn through. The author draws unfavourable conclusions to forcible rectification, and recommends an apparatus of his own for keeping up continuous traction.

Dr. Phelps[3] says that this practice is as old as the history of medicine, having been revived and practised by Hippocrates 500 B.C., and again in the sixteenth century by Pere. And now Chipault and Calot contend that all cases of Potts' curvature can be cured by forcible adjustment under an anæsthetic. But they are anticipated in these contentions by Dr. B. E. Hadra, of Galveston, Texas. In the *Times and Register*, May 23rd, 1891, in speaking of his case of spinal fracture which he straightened and held in place by wiring the spinous processes, he suggests the treatment of spinal disease by the same method. He advocates this as soon as displacement of the bone is noticed, and in cases where full kyphosis is present to anæsthetise the patient and wire together the spinous processes, providing the spinal column can be straightened by force. He operated on his case, and straightened a fractured spine by wiring together the spinous processes, December 22nd, 1890. So that the modern revival of this ancient practice is truly American and not French.

The history of the operation may be summed up as follows: Chipault says "that unless the spinous processes are wired together nearly all the cases will relapse." Calot reports 204 cases of forcible reduction with no accident and no immediate deaths. Two died within three months from meningitis and pneumonia. There were six cures of paralysis in eight cases, and one case resulted in partial paralysis. Monod says that Calot's statistics are too good. Chipault reports many relapses. Jones and Tubby report eleven operations with six cases of complete rectification and five partial at the time of operation. Lorenz has one case of paralysis and relapse following the operation. Jonnesco had three deaths and thirteen relapses. Pean, Vincent, Lorenz, and Tausch have all reported relapses. Ducroquet has shown radiographs which reveal bony repair of the gap in the bodies of the vertebræ. Calot claims that the gaps fill in with new bone. Ridlon was the first to operate in America, and reports seven cases. Gibney presented two cases at the orthopædic section of the Academy in March, 1898, and the author presented one. Professor Lorenz reports a case of permanent paralysis following the operation. On the cadaver Monod ruptured an abscess into the mediastinum. Lorenz and Monod severely criticise the operation; Calot is enthusiastic; Chipault extremely cautious and conservative, and many of the profession in Europe who have witnessed the work are enthusiastic. Many of the surgeons of London have expressed themselves in doubt, and desire that opinions will not be expressed until final results are shown.

The operation is certainly not a dangerous one. It is not intended to cure Potts' disease, but a deformity incidental to it; therefore the operation is justifiable, as much as that for correcting deformities of the hip joint, and the treatment of hip-joint disease is begun by overcoming the deformity alone. This operation will relieve paralysis due to bone pressure, but it can in no way relieve paralysis due to pressure myelitis, the result of tubercular invasion of the spinal cord. When there is extensive destruction of the bodies of the vertebræ, resulting in a large kyphosis, reproduction of the bodies of the vertebræ is out of the question. Such spines could only be held in a straight position by resorting to Hadra's operation of wiring the spinous processes. If large abscesses are present systemic infection from rupturing the abscess in adjacent tissues and further bone invasion would probably result.

The author draws the following conclusions. That ankylosed spines of old standing, with a large kyphosis, should not be operated upon. Cases with large abscesses should be avoided. Cases of osteomyelitis after the abscess has been evacuated would promise more than tubercular cases. Cases in which good results may be expected are: Beginning kyphosis and of recent standing, and unattended with abscess; where abscess had already discharged in which a small kyphosis was present; beginning paralysis and even advanced paralysis not due to invasion of the spinal cord by the disease. Cases of lumbar disease are more favourable than dorsal. The author thinks that the indiscriminate work which will be done in the future by individuals incapable of selecting their cases or actuated by other motives, will relegate the operation to the obscurity in which it has slept for more than 2,000 years.

**Lateral Deviation of the Spine and Pes Canis in Friedreich's Ataxia.**—Dr. W. R. Townsend[4] presented a boy, 12 years of age. He had an unsteady gait and inability to keep from falling if pushed. For the past seven years he had had frequent pain in the knees. Lateral curvature of the spine appeared three years ago, and had steadily increased, a long curve to the right extending from the ninth dorsal vertebra downward, with rotation. A plaster of Paris corset had been applied with moderate suspension. There was pes canis but no equinus. The gait was markedly ataxic. Standing with feet separated and eyes closed there was swaying of the body. The patellar reflexes were lost. Speech was slow. There was mistagmus, but no Argyle-Robertson pupil. Dr. J. Collins said it was a clinically typical case. In addition to disease of the posterior columns there was sclerosis of the lateral parts of the cord, including the direct cerebellar tracts shown by persistent efforts of the patient to balance himself, and producing the peculiar condition found in every case heretofore undescribed, aptly named the "fork-prong" condition of the extensor tendons, the feet being continually on the balance. A potent agent in restoring the function of the muscles was the re-education of the extremities. The patient might be so taught that in a few months he would be able to walk into the room without perceptible disturbance of gait.

**The Modern Treatment of Congenital Dislocation of the Hip.**—Hoffa,[5] in a pamphlet recently published, describes an operation of his own, which consists in the reposition of the abnormally-placed head of the femur, previously exposed by the knife, into a newly-excavated cotyloid cavity. The steps of this operation are a vertical incision of the skin and soft parts along the anterior border of the great trochanter; free incision of the capsule and exposure of the displaced head of the femur; enlargement by the use of the sharp spoon of the rudimentary cotyloid cavity, so that it is made deep and wide enough to receive the head of the femur; replacing of the head of the femur into the enlarged cotyloid cavity, a step in the operation which in many patients, and especially in those above the age of childhood, is attended with much difficulty, often necessitating forcible extension of the limb, and occasionally tenotomy of the adductors. Great importance is attached to the after-treatment, the details of which are fully described. Hoffa has performed this operation in 200 cases, in six of which death was a direct result of the treatment. Whilst advocating his own method, he acknowledges that it cannot restore all the conditions of a sound and normal joint. Shortening of the limb must persist, and a complete range of movements cannot be attained. The condition of the limb with regard to form, disposition and functional capacity resembles that met with in coxa vara. The advantages to be derived from a successful operation are lengthening of the limb, diminished laxity and mobility of the head of the femur, and improved position and increased activity of the gluteal muscles. The younger the patient the better are the prospects from this operation. The best time for its performance is from the third to the eighth years of life. It is contra-indicated in subjects beyond the age of 10, in whom, in cases of bilateral dislocation, Hoffa would remove both femoral heads.

**Traumatic Epiphysial Separation of the Head of the Femur, with its Relation to Coxa Vara.**—Sprengel[6] gives two cases that closely simulated coxa vara. In both cases the epiphysis was displaced downward and had united in a false position that hindered movement and produced symptoms so much like coxa vara that the true condition was not diagnosed until after operation. The head of the femur was resected, leaving the great trochanter and part of the neck. There was increased movement in the limb, but neither patient could stand on the affected limb, and they were compelled to use crutches. The author finds that recent investigation has shown that traumatic separation of the epiphysis is much more frequent in the head of the femur than was formerly supposed, and recommends that the condition found present in the epiphysis should not be lost sight of. He believes that resection and removal of the head of the bone is preferable to any other treatment.

[1] Wiener Klinik, 1 Heft, 1899. [2] Arch. für klin. Chir., Band lvii., p. 485. [3] The Post Graduate, Feb., 1899, p. 102. [4] Med. Times and Register, 1899, p. 191. [5] Brit. Med. Jour., Feb. 25, 1899. [6] Arch. für klin. Chir., Band lxvii., 4 Heft.

